# Evaluation of Scissor Glide Characteristics Through Surgeons' Subjective Assessment: The Application of Nitriding in Scissor Hardening Tests

**DOI:** 10.7759/cureus.52402

**Published:** 2024-01-16

**Authors:** Gaku Ota, Mikio Shiozawa, Jun Watanabe, Yoshitaka Maeda, Kosuke Oiwa, Jun Mizuno, Naohiro Sata, Hiroshi Kawahira

**Affiliations:** 1 Department of Surgery, Division of Gastroenterological, General, and Transplant Surgery, Jichi Medical University, Shimotsuke, JPN; 2 Department of Surgery, Tochigi Medical Center Shimotsuga, Tochigi, JPN; 3 Medical Simulation Center, Jichi Medical University, Shimotsuke, JPN; 4 Department of Information and Management Systems Engineering, Nagaoka University of Technology, Nagaoka, JPN; 5 Program on Smart and Sustainable Manufacturing, Academy of Innovative Semiconductor and Sustainable Manufacturing, National Cheng Kung University, Tainan, TWN

**Keywords:** ergonomics, nitriding, surgical scissors, subjective resistance value, surgeons' subjective assessment

## Abstract

Introduction: In robotic surgery, studies on providing tactile feedback to users are ongoing. However, the accuracy of the subjective sensations of surgeons, as users, has been largely unassessed. This study aimed to assess the validity of surgeons' subjective evaluations of scissors resistance through interindividual, inter-surgeon, and objective evaluations. Furthermore, in this study, we explored the possibility of using nitriding to increase the hardness of the scissors and assessed changes in subjective resistance values before and after nitriding using this approach.

Method: Five surgeons conducted validation of five curved surgical scissors (145 mm; Arakawa Seisakujyo Co., Ltd., Tokyo, Japan) and assessed their subjective resistance using a scale from 0 to 10, where a rating of 10 signified significant resistance impeding the scissors' closure. The temporal changes in subjective resistance values, from maximum open to close, were graphically recorded. To demonstrate the reproducibility of subjective resistance values, the subjective resistance values of the same control scissors were measured at intervals of at least two weeks, and the correlation coefficient was calculated. To analyze the closing characteristics of subjective resistance values between different pairs of scissors, the effect of scissor type and scissor closure position was compared as two independent variables using a two-factor analysis of variance. A comparative evaluation was conducted to assess the frictional properties of scissors after nitriding, comparing the subjective assessment by surgeons with the objective assessment using a digital force gauge.

Results: The correlation coefficient of subjective resistance values measured by surgeons demonstrated a high reproducibility of 0.746. A two-factor analysis of variance conducted on subjective resistance values demonstrated the presence of a primary effect for the sample factor (scissors), as well as for the position factor (closing process), with the additional observation of the interaction between these two factors. The results from the two-factor analysis of variance above provide evidence supporting the validity of the subjective resistance measurements. There was a significant increase in subjective resistance after the nitriding process. The graph of subjective resistance values and objective resistance values showed similarity.

Conclusions: The surgeons' subjective assessment of scissors resistance showed high reproducibility and validity, as evidenced by distinguishable differences in scissor movement interactions and pre- and post-nitriding resistance. Further studies are warranted to expand on these findings.

## Introduction

Medical scissors have been in use since the 15th century, and it is safe to say that, among all surgical instruments, scissors are perhaps the most frequently used - prior to, during, and after operation [1]. While numerous innovative instruments have been devised to cater to the evolving domains of laparoscopic and robotic surgery, many retain semblances to the rudimentary scissor structure, epitomized by their pivotal grip and characteristic finger-insert rings. Scissors constitute the foundational archetype of surgical instruments, rendering them indispensable in laparoscopic and robotic surgical endeavors.

Previous studies assessed the resistance values of surgical scissors through various objective and subjective methods [2-5]. Quantitative haptic feedback to surgeons has catalyzed the advent and refinement of haptic feedback systems, revolutionizing robotic surgery and its associated simulations [6,7]. However, the accuracy of the subjective sensations of its primary operator - the surgeon - remained predominantly unassessed.

An ancillary challenge that plagues the utility of scissors pertains to their susceptibility to wear and tear over time. Nitriding, conventionally employed to enhance the wear resistance, fatigue durability, and corrosion resilience of components in industries such as automotive and marine, is seldom utilized in the fabrication of surgical paraphernalia. While nitriding increases material hardness, there are concerns that it may simultaneously increase resistance [8]. 

This study aimed to assess the validity of surgeons’ subjective evaluations of scissors resistance through inter-individual, inter-surgeon, and objective evaluations. In addition, we explored the possibility of using nitriding to increase the hardness of the scissors and assessed changes in subjective resistance values before and after nitriding using this approach.

## Materials and methods

**Figure 1:**
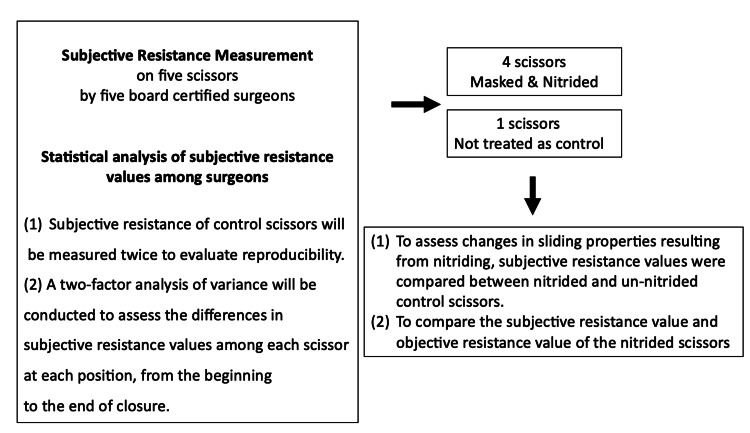
Schema of this experimental overview

The experiments were conducted in accordance with the attached schema (Figure 1). The study encompassed four processes: subjective resistance measurement on five scissors, statistical analysis of subjective resistance values among surgeons, low-temperature plasma nitriding treatment and masking, and measurement of subjective and objective resistance values after nitriding.

Subjective resistance measurement on five scissors

**Figure 2:**
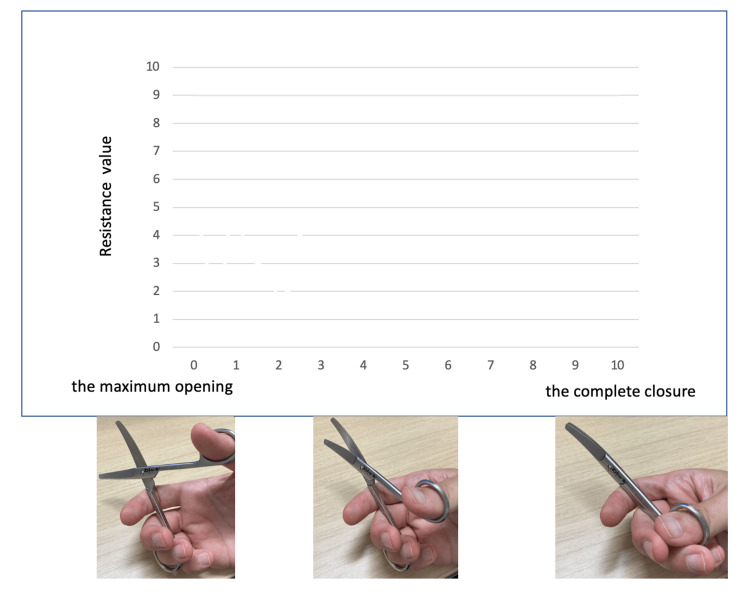
Resistance value entry form Resistance value “10” refers to the point at which the measurer perceives that further stiffness would prevent closure

Five board-certified surgeons subjectively evaluated the resistance values while closing the same five curved surgical scissors (145 mm; Arakawa Seisakujyo Co., Ltd., Tokyo, Japan). The surgeon evaluates the hardness throughout the closing process and subjectively depicts it as a graph in “Resistance value entry form.” The horizontal axis represents the position score, ranging from 0 (the maximum opening of the scissors with the blades just starting to intersect) to 10 (the complete closure of the scissors). The vertical axis represents the resistance value encountered during the closure of the scissors, with a value of 10 indicating an extreme level of hardness. A score of 10 indicates the rigidity level where the surgeons determine that the scissors cannot be further constricted, regardless of the scissors’ intrinsic stiffness (Figure 2).

Statistical analysis of subjective resistance values among surgeons

We initiated our study by selecting five pairs of scissors. Among five pairs of scissors, one pair was designated as a control. After a washout period of at least two weeks, subjective resistance values of the control scissors were measured again to assess the correlation analysis. We performed correlation analysis to evaluate the reproducibility of subjective resistance assessments by surgeons. To scrutinize the potential influence of the two independent variables, namely "scissor type" (designated as the sample factor) and "scissor closure position from the beginning to the end" (referred to as the position factor), on variations observed in the closure characteristics of subjective resistance values (the designated response variable), a rigorous two-factor analysis of variance was employed. We also searched for interaction if the two factors independently affect the subjective resistance value. Subsequently, back-testing was employed to examine the sample factor and determine if there were any significant differences.

To analyze any changes in the subjective resistance values before and after nitriding, the surgeon conducted another round of measurements to determine the subjective resistance values. A two-factor analysis of variance was employed again. The analysis was executed using R version 4.0.2 (The R Foundation for Statistical Computing, Vienna, Austria). The threshold for significance was *P*<0.05.

Low-temperature plasma nitriding treatment and masking

The scissors, except the control scissors, underwent low-temperature plasma nitriding. This process, conducted at temperatures below 500°c, utilized a plasma generator employing a self-excited oscillation method. This method combines the application of radio frequency power in the radio frequency band with a direct current bias (Table 1).

**Table 1:** Nitriding Condition DC, Direct Current; RF, Radio Frequency.

Scissors Number		H_2_(ml/min)	N_2_(ml/min)	DC bias(V)	RF(V)	Reactor temperature(℃)	Nitriding time(h)
１	No masked	10	50	300	230	420	4
２	Masked in 3mm	10	50	400	230	420	4
３	Masked in 0.5mm	10	50	400	230	420	4
４	Masked in 0.5mm	10	50	400	230	420	4

To assess the effect of nitriding extent on the sliding property, Scissors 1 was completely nitrided without any masking. Scissors 2, 3, and 4 had masking applied using aluminum foil to protect areas other than the beveled edge and inside blade surfaces. This masking strategy helps avoid excessive nitriding and the resulting reduction in sliding performance. The hardness after nitriding was measured using a micro-Vickers hardness tester. The hardness of the nitrided and masked areas was analyzed using the Wilcoxon rank-sum test to determine if there was a significant difference.

Measurement of subjective/objective resistance values after nitriding

**Figure 3:**
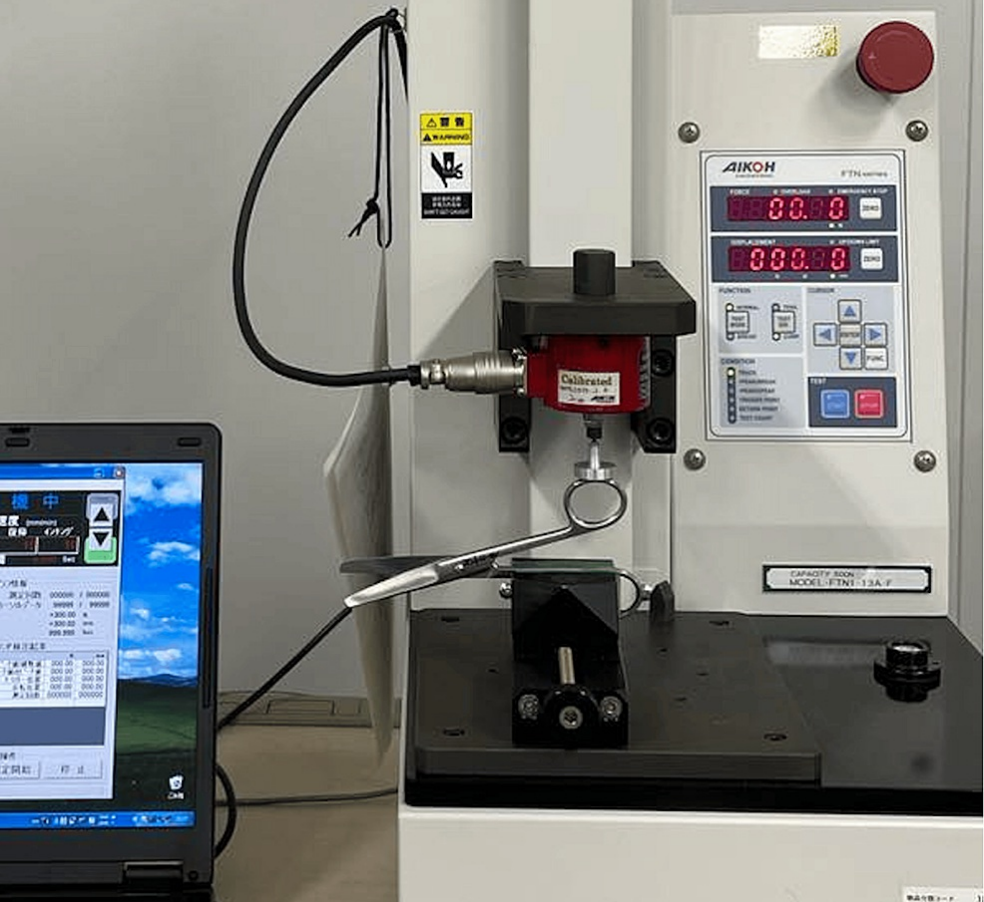
Digital force gauge and small tabletop testing machine. The surgical scissors were unfolded, and one of the handles was clamped horizontally in a vise.

To assess the nitriding effects, subjective resistance measurements were conducted by a surgeon on four nitrated scissors and one control scissors. Changes in resistance values before and after nitration were examined using a two-factor analysis of variance. To compare the subjective resistance values after nitration, the scissors’ objective resistance values were measured using a digital force gauge. The objective resistance measurements were conducted using a small tabletop testing machine (model FTN1-13A-F, manufactured by AIKOH engineering, Nagakute, Japan) and a digital force gauge of 20 N (also manufactured by AIKOH engineering). The surgical scissors were opened, and one handle was secured horizontally using a vise. When the surgical scissors were fully opened, the handle descended due to its weight until it reached a position where the weight of the handle balanced with the resistance of the scissors, at which point the handle came to a stop. This position was considered the initial position (Figure 3).

The scissors handle was compressed at a speed of 10 mm/min until the scissors were completely closed, and the resistance value and time to close the scissors fully were measured. The resistance values (N) were measured twice for each pair of scissors, and graphs were created with the average resistance values on the vertical axis and the measurement time (seconds) on the horizontal axis. The horizontal axis was adjusted so that the moment when all five pairs of scissors were fully closed corresponded to 0 seconds.

## Results

Surgeons' subjective ratings exhibited a high level of reproducibility

**Figure 4:**
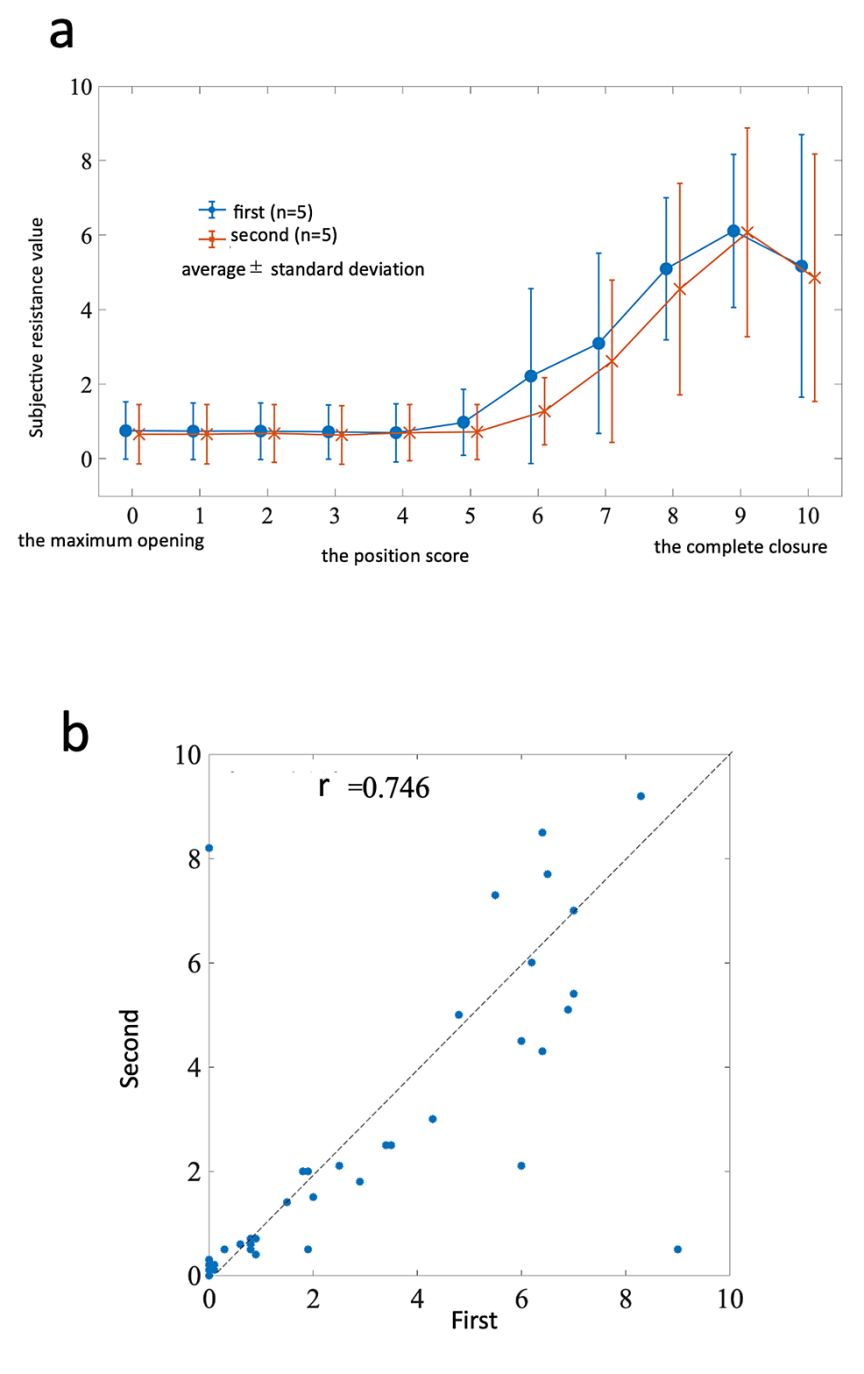
a) The mean and deviation of first and second subjective resistance to control scissors. b) The correlation between first and second subjective resistance to control scissors. b) The correlation coefficient = 0.746

The mean and deviation of the subjective resistance values of the control scissors, as measured by five surgeons, were analyzed twice (Figure 4a). The resistance value increased starting from position score 5, which coincided with the portion where the curvature of the surgical scissors began. A scatter plot was constructed to represent the subjective resistance of each subject during the first session on the horizontal axis and the subjective resistance during the second session on the vertical axis. The correlation coefficient, determined to be 0.746, indicated a strong correlation. Thus, the measurement of subjective resistance by the experienced surgeons was deemed highly reproducible (Figure 4b).

**Figure 5:**
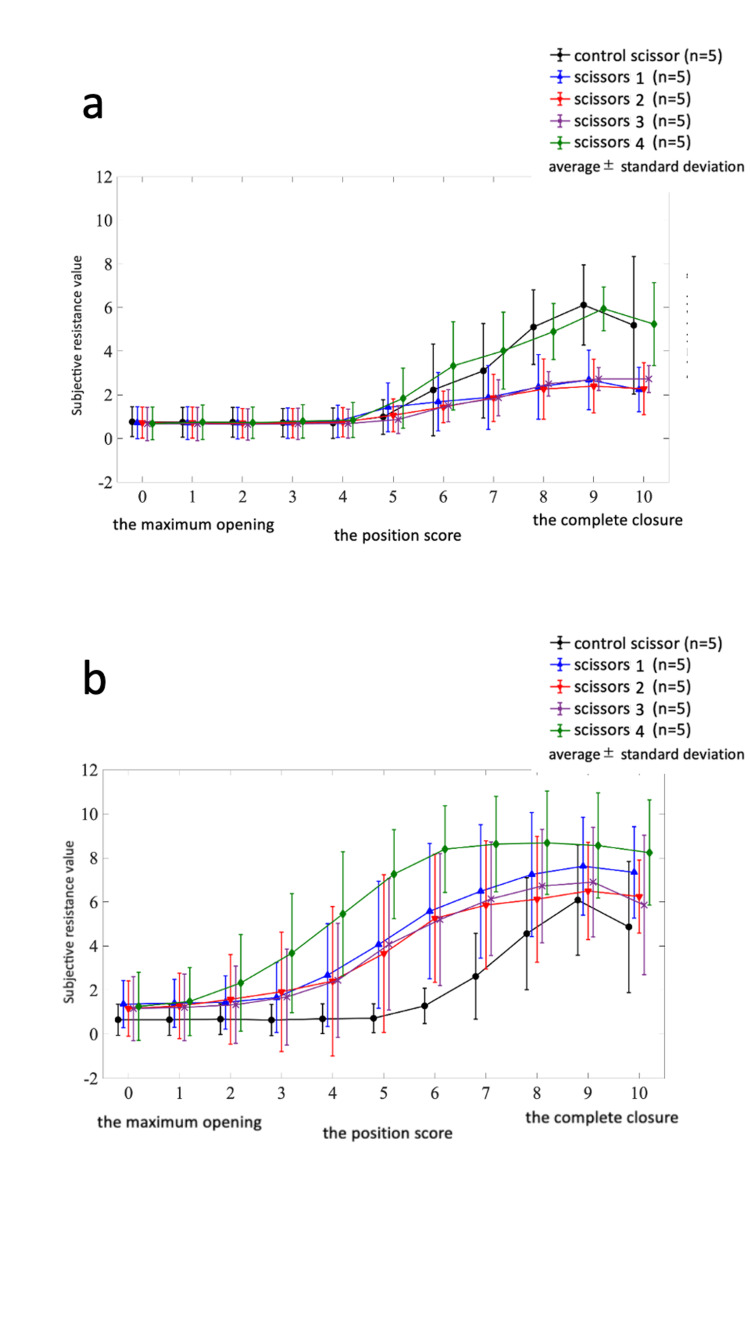
The mean subjective resistance of scissors; a) The first measurement prior to nitriding. b) The second measurement following nitriding. a) The first subjective resistance measurements were conducted with a two-factor analysis and a back-test prior to nitriding. Two-factor analysis: Sample factor, main effect present (*p* < 0.0001); Position factor, main effect present (*p* < 0.0001); Interaction, present (*p *= 0.0131) Back test: Combinations with significant differences between control and other samples; Control Scissors > Scissors 1 (*p* = 0.0002), Control Scissors > Scissors 2 (*p* < 0.0001), Control Scissors > Scissors 3 (*p* = 0.0001) Other combinations with significant differences. Scissors 4＞Scissors 1 (*p* < 0.0001, Scissors 4＞Scissors 2 (*p* < 0.0001), Scissors 4＞Scissors 3 (*p* < 0.0001） b) Scissors 1 to 4 exhibited a significant increase in subjective resistance following nitriding (main effect on condition factor: *p* < 0.0001). No change in subjective resistance was observed in the control scissors (*p* = 0.4447).

A two-factor analysis of variance was conducted on the first subjective resistance measurements performed by surgeons prior to nitriding. The results indicated the main effect of the sample factor (per scissors) (F(4) = 12.5008, *p* < 0.0001, *partial η2* = 0.0847), the main effect of the position factor (F(10) = 25.4379, *p* < 0.0001, *partial η2* = 0.4309), and the interaction between these two factors (F(40) = 1.6478,* p* = 0.0131, *partial η2* = 0.1117) (Figure 5a). The findings from the two-factor analysis of variance showed that scissor type (the sample factor) and scissor closure position (the position factor) serve as independent variables on the variations observed in the closure characteristics of subjective resistance values (the response variable). Furthermore, the analysis substantiates the existence of a statistically significant interaction between these two factors. The back-testing results for multiple comparisons among the samples revealed significant differences in the overall position score (ranging from 0 to 10) between the control scissors and scissors 1, 2, 3, as well as scissors 4 and scissors 1, 2, 3. Likewise, significant differences were observed between the control scissors and scissors 1, 2, 3 and scissors 4 and scissors 1, 2, 3 in the shear closing position scores of 8, 9, and 10.

Nitriding offers enhanced hardness, while masking prevents nitriding and significantly increases subjective resistance

The hardness of the nitrided areas and the masked areas on the front and back of the blade were measured using Vickers Hardness (HV) (Table 2). The nitrided parts of the four scissors exhibited a hardness of 1,214.1±120.2 HV, while the masked parts had a hardness of 584.5±26.8 HV, indicating a significant increase in hardness in the nitrided parts (*p* < 0.0001).

**Table 2:** Hardness following nitriding HV, Vickers Hardness.

		Front (HV)		Back (HV)	
		Nitrided	Masked	Nitrided	Masked
Scissors １	total nitrided	1291		1152	
Scissors ２	masked	1278	562	1130	608
Scissors ３	masked	1334	558	1119	558
Scissors ４	masked	1393	626	1016	595

Prior to nitriding, scissors 4 exhibited a subjective resistance transition similar to that of the control scissors, whereas scissors 1,2, and 3 displayed lower resistance than the control scissor. However, after nitriding, all resistance values increased from the beginning of closing (position score 0). Scissors 1, 2, and 3 demonstrated higher resistance values than the control scissor, while scissors 4, which already had high resistance values prior to nitriding, exhibited even higher resistance values.

The two-factor analysis of variance revealed no change in the subjective resistance of the control scissors during the second measurement (*p* = 0.4447), maintaining a strong correlation with the first measurement, as mentioned above. However, scissors 1, 2, 3, and 4 showed a significant increase in subjective resistance following nitriding (*p* < 0.0001) (Figure 5b).

Subjective resistance values and objective resistance values exhibit similarity

**Figure 6:**
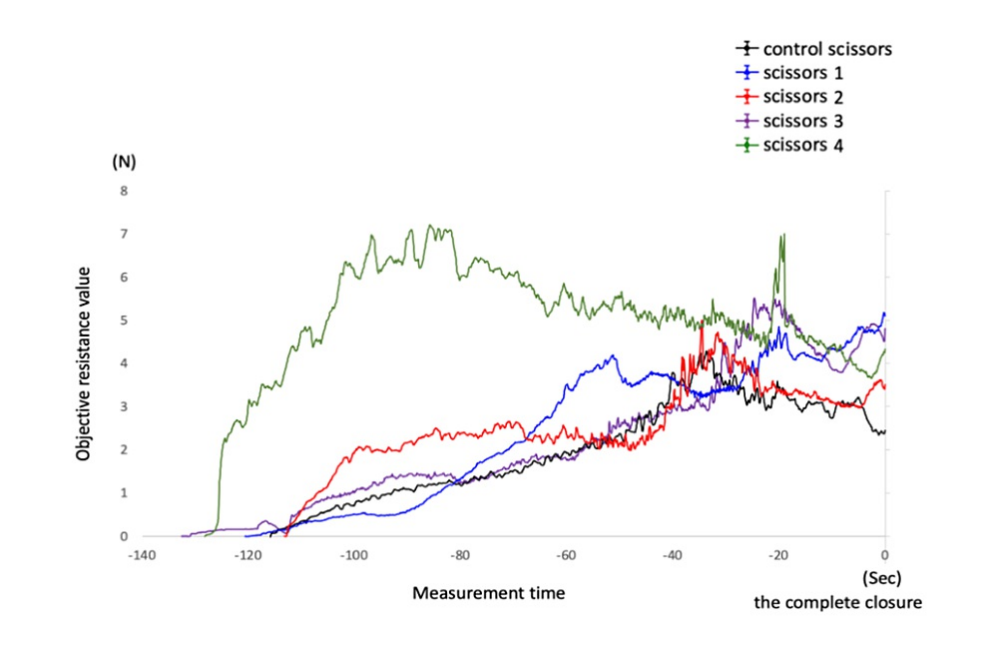
An Investigation into Scissors' Objective Resistance: Evaluating Measurements Post Nitriding Treatment

The graphs of subjective (Figure 5b) and objective resistance values (Figure 6) were compared. Within the two graphs, compared to the control scissors, scissors 1, 2, and 3 showed a slight increase in resistance values, whereas scissors 4 exhibited a notable increase in resistance values. Despite differences in the units of the vertical and horizontal axes between the two graphs, a common purpose of measuring scissor resistance values showed a notable similarity.

## Discussion

This study demonstrates that surgeons’ subjective assessments of scissor resistance exhibit a high level of consistency among individuals. Using this reproducibility, this study accurately assessed variations related to scissor types, the closing process, and interactions in the subjective resistance values of surgical scissors. Furthermore, there was clear evidence that the surgeons’ subjective and objective assessments of scissors were consistent after nitrification increased resistance. Sensory-based techniques such as auscultation, palpation, and visual examination stand as the cornerstone of diagnostic modalities. In the field of pediatric medicine, there have been previous examinations of the reliability of maternal subjective assessment of fever [9,10]; however, even after a long period, its reproducibility has not been established [11]. Scant research has delved into appraising the replicability of palpation skills among medical practitioners. Even in contemporary literature, the evaluation of a patient's abdominal condition through palpation remains a formidable clinical skill to replicate due to its heavy reliance on tactile perceptions and the requisite responsiveness from patients [12]. To the best of our knowledge, this study is the first to conduct a statistical analysis of subjective assessment of tactile examination by medical doctors and compare these outcomes with objective resistance metrics.

This study is novel in that two-dimensional subjective resistance values during scissors use were measured and evaluated between individuals and multiple surgeons over time. A thorough literature review revealed an absence of studies on this specific topic. Although there are existing studies that explore the relationship between surgeons and scissors. Greenish et al. developed a cutting simulator and assessed whether surgeons could subjectively distinguish between real organs and virtual tissue on the simulator [2]. Berguer and Harper conducted a unidimensional evaluation using a simple rating for factors like difficulty and cutting ability during scissor use without conducting statistical analysis [4,5]. Shimomura et al. conducted to objectively measure the fatigue experienced by surgeons when using scissors by employing electromyography to monitor muscle activity [13]. In comparison to the studies mentioned, adopting the time-intensity analysis with two-dimensional values in our research allowed us to statistically demonstrate the reproducibility of surgeons' perceptual values.

The time-intensity analysis differentiates itself from conventional descriptive analysis by examining changes in the perception of an attribute over time. Conventionally, this method has been adopted as an assessment technique for sensory values, such as taste, within the realm of food-related studies [14,15]. When applied to medical and engineering systems, this method yielded results that consistently demonstrated high levels of reproducibility and reliability. This was achieved despite its straightforward reliance on the surgeons’ tactile perception. We believe our approach holds potential for future studies in tactile feedback and can be applied accordingly.

In advanced surgery, scissors are vital; however, in developing countries, problems like difficulty sharpening and inconsistent supply can arise. Enhancing the durability of scissors in any medical environment is of great interest. All the nitrided scissors, including those masked, had significantly increased resistance compared to before nitriding. It is possible that slight deformation or an initial increase in the coefficient of friction could occur despite the use of low-temperature nitriding processes. Moving forward, alternative hardening methods, such as diamond-like carbon, will be considered.

This study had some limitations. This study measured resistance by closing the scissors without cutting any actual tissue or material, referred to as a “blank run,” to prevent the blade from dulling or experiencing changes in sharpness due to tissue or material adhesion while evaluating the same pair of scissors by multiple individuals multiple times. However, blank runs in experiments are often used as control runs or baseline measurements [2]. Additionally, our study is limited by the small sample size, involving only five scissors and five participating surgeons.

## Conclusions

This study is considered the first to scientifically demonstrate the high reproducibility of tactile feedback in surgeons through surgeons' subjective evaluations of scissors resistance. While nitriding the scissors allowed for blade reinforcement, we were able to prove a significant increase in resistance values following nitriding using subjective measurement methods.

The high reproducibility of tactile feedback in surgeons is believed to be crucial foundational data for future developments in other medical devices.
